# The Health of Firefighters Deployed to the Fort McMurray Fire: Lessons Learnt

**DOI:** 10.3389/fpubh.2021.692162

**Published:** 2021-11-11

**Authors:** Nicola Cherry, Jeremy Beach, Jean-Michel Galarneau

**Affiliations:** Division of Preventive Medicine, University of Alberta, Edmonton, AB, Canada

**Keywords:** Fort McMurray fire, firefighters, particulate exposure, respiratory, mental ill-health

## Abstract

**Introduction:** Firefighters were working in exceptionally difficult conditions during the Fort McMurray/Horse River fire in May 2016.

**Methods:** From mid-May, we recruited firefighters from 13 fire services as they returned from the fire. In October 2016 we extended recruitment to all Alberta-based firefighters deployed to the fire. In December 2017–May 2018 we sent a first online follow-up: this concentrated on mental health supports. The second follow-up, in October 2018–January 2019, included screening scales for respiratory ill-health and PTSD. All three contacts included visual analogue scales for chest symptoms and the Hospital Anxiety and Depression Scale. We estimated exposure to PM_2.5_, and calculated an exposure mitigation index from reports of respiratory protective equipment (RPE).

**Results:** We recruited 1,234 firefighters and examined the relation of respiratory symptoms to estimated particulate exposure. The relation was strong immediately post fire but weakened over time. We found less chest tightness and cough in those whose RPE in the first week mitigated exposure by at least 10%. We examined the relation between particulate exposure and mental ill-health from screening questionnaires and found those with high exposure (reflecting the ferocity of the fire) had poorer mental health scores. Firefighters reporting their “worst moment during the fire” was life threatening were more anxious at second follow-up. Overall both anxiety and depression scores increased at successive contacts, more so in those with mental ill-health recorded in physician billing records before the fire.

**Discussion:** The results from this study overall suggest on-going fire-related health effects in a substantial minority of firefighters, similar to those reported in the longitudinal follow-up of firefighters after the collapse of the World Trade Centre. Self-reports of both respiratory symptoms and mental ill-health were strongly related, soon after the fire, to estimated particulate exposures. Anxiety increased over time since the fire in those who felt their life or safety had been threatened, underlining the need for ongoing support. Our conclusions about the benefits of rapid research relate particularly to the collection of biomarkers of exposure as quickly and widely as possible, and the establishment of a nominal list of participants before they are too widely dispersed.

## Introduction

The Fort McMurray/Horse River fire overwhelmed the conurbation and surrounding areas on May 3rd 2016 (1). It was immediately apparent that firefighters were being exposed to exceptionally difficult conditions and on 5th May we discussed a study of the effects of the fire on firefighters' respiratory health with the Occupational Health and Safety team at Alberta Employment (now Alberta Labour). By chance we had taken delivery that week of a clinical mobile laboratory (funded by the Canadian Foundation for Innovation) equipped to carry out respiratory function testing and to handle biological samples. With immediate collaboration from the Strathcona fire service we developed a protocol and exposure questionnaires and on the 16th May began our assessment of Strathcona firefighters who had been deployed to the fire.

This rapidly developed protocol formed the basis for all the work that followed and reflected the three areas that seemed of key importance: assessing effects on respiratory function (2–4) and mental health (5, 6) and making the best possible estimates of exposure. Early in the fire we were able to collect samples (urine and blood) to look for biological markers of exposure (7, 8) and to develop a questionnaire that allowed us to create exposure algorithms incorporating data from environmental monitoring and satellite imagery. There was no access for researchers to the fire area during the first weeks of the fire and we were dependent for environmental sampling on data collected by others. We assessed the firefighters as soon as we could after they had returned from a rotation.

The work we have done with the firefighters deployed to the Fort McMurray fire has aimed to identify exposures and work practises that could be modified to reduce harm in future conflagrations and to help in the recognition of fire-related ill-health. To do that successfully we needed to rely as much as we could on objective measures of exposure and effect. We have previously given detailed results on many aspects of the study (7–12) but here we want to consider particularly the firefighters' perceptions of their experiences and how these relate to their ongoing ill-health. We describe the evolution of respiratory symptoms with time since the fire and the nature of the respiratory conditions that do not resolve. We look also at the part played by respiratory protective equipment (RPE) in preventing respiratory ill-health in those exposed during the fire. Finally, we address the complex relation between firefighters' own perception of their worst moments during the fire and their mental health many months post-fire.

## Methods

### Data Collection

In Phase 1 of the study, from May to October 2016, we visited 12 structural and one industrial fire service. At each we talked to the firefighters informally about their experiences during the fire and then asked them to consent to completing a recruitment questionnaire about their exposures during the fire and their health. We also asked if they would consent to linkage with the Alberta Administrative Health Data Base (AHDB). At two fire services we collected blood samples to analyze for inflammatory markers and at three we collected urine samples, to look for markers of exposure to polycyclic aromatic hydrocarbons (PAHs). At all 12 of the structural fire services we carried out spirometry to assess lung function after the fire. All the Phase 1 recruitment was face-to-face.

For Phase 2 we aimed to approach all firefighters based in Alberta who had been deployed to the Fort McMurray/Horse River fire. We had two sources to identify these. The provincial premier had requested that a list be drawn up of all those who had been deployed from structural and industrial fire services, so that they could be thanked individually. This showed the names and fire service and using this we were able to send letters (through the fire chief) to everyone on the list inviting them to take part and to complete the recruitment questionnaire online. For wildland firefighters an operation list had been drawn up with the names and management area of each wildland firefighter deployed and again we were able to contact many though their area manager. Neither list was fully inclusive: some fire services did not submit names to the premier's list and a high proportion of wildland firefighters worked only during the fire season and could only be contacted when and if they returned to employment. We increased recruitment among wildland firefighters by attending base camps when part-timers returned to employment in 2017 and 2018. We also made a concerted attempt to maximise enrolment among those based in the Fort MacMurray area by face to face meetings with structural and industrial fire fighters in the summer of 2017. Again, there was emphasis on getting factual data about experiences during the fire and consent, where forthcoming, to link to the AHDB.

**Figure 1:**
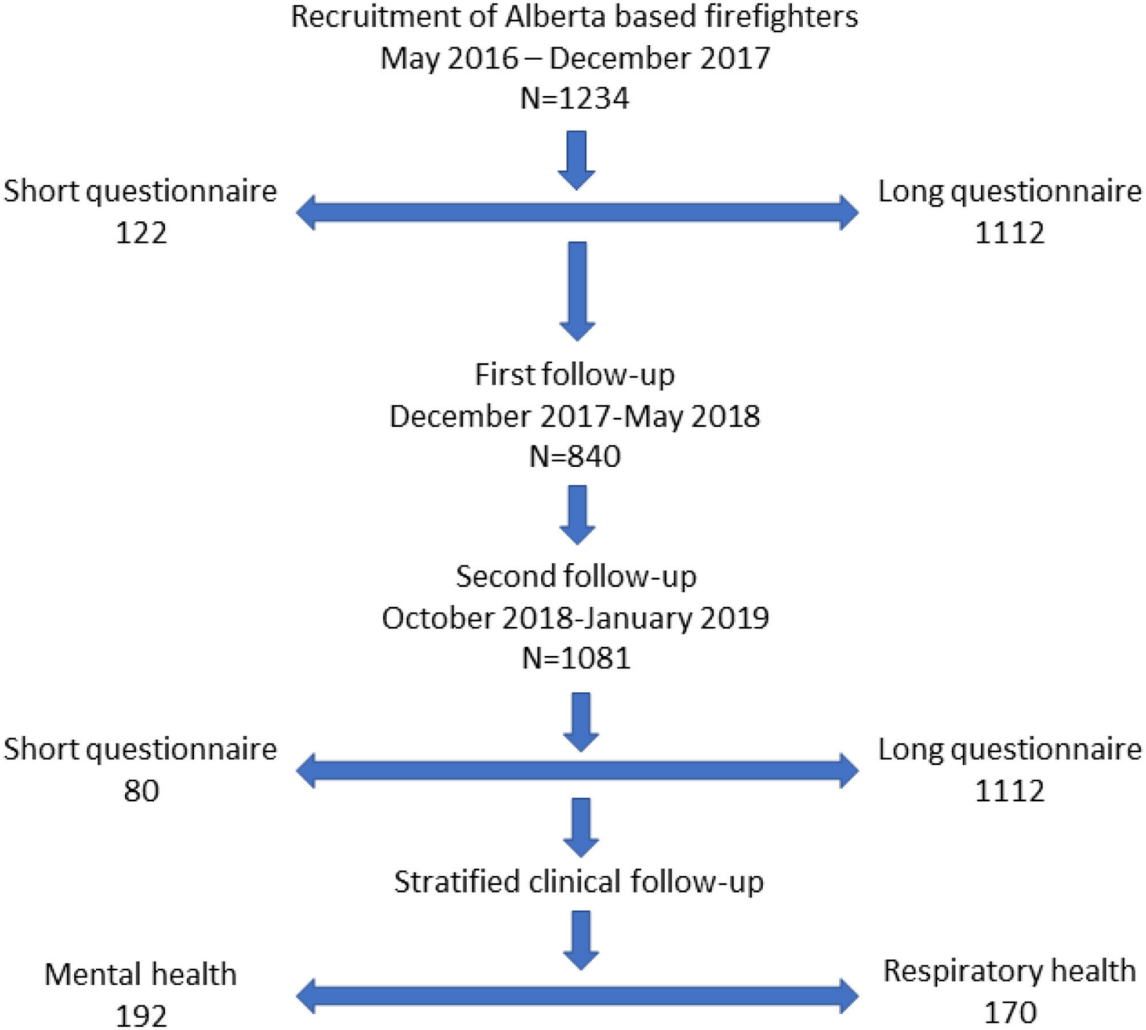
Response of firefighters at each contact.

The first follow-up was an on-line questionnaire in December 2017 to May 2018 (Figure 1). This asked, particularly, about the types of mental health supports that had been available through the employer, before, during and since the fire. Those who had very recently completed the recruitment questionnaire were not approached for this follow-up. The second follow-up was October 2018–January 2019: everyone who had completed the recruitment questionnaire was requested to complete this and, for those very reluctant, a short questionnaire was completed on-line or by telephone. The full questionnaire included screening questionnaires for anxiety and depression [using the HADS questionnaire (13, 14) and for PTSD with the PCL-5 (15)], and the European Community Respiratory Health Survey (ECRHS) (16) to screen for respiratory ill-health.

The final phase was to carry out clinical assessments using stratified random samples where the invitation to undergo assessment depended on scores on the screening questionnaires in the second follow-up. The respiratory assessment comprised lung function testing, a methacholine challenge test (to look for airways hyperreactivity) and a CT scan of the chest to assess bronchial wall thickening and to rule out other causes of lung dysfunction such as fibrosis. The psychiatric assessment took the form of a Structured Clinical Interview to see if the mental ill-health met the criteria for a particular diagnosis on the DSM-5 (17). This type of interview is known as a SCID-5. These assessments were important in that it allowed us to talk about the prevalence of objectively measured ill-health associated with attendance at the fire.

We collected other data also. In May 2017–January 2018 we interviewed fire chiefs or, for wildland firefighters, area managers to ask them about the mental health provision and support offered to the deployed firefighters, before, during and after the fire. We also asked the firefighters if they had had spirometry as part of health monitoring by the fire service, and if so, whether they consented to us obtaining copies of the records before and since the fire. We requested consent to get information from the Workers Compensation Board (WCB) of any fire-related claims. Finally, we asked the Alberta AHDB to match each firefighter (who had given consent to be linked) with 5 people matched by age (±3 years), sex, geographic area and use of the health services in the 12 months before the fire. In Alberta, to be paid for services, a physician has to record at least one diagnosis for each claim. For this set of firefighters and community controls we were able to look at the diagnosis for all physician claims in the 3 years before the fire and 2 years after, to see if the pattern diverged for the firefighters compare to controls. We were also able to use the information contained in billing records to document whether the firefighter had respiratory or mental ill-health, as recorded by a physician, in the 3 years before the fire.

### Definitions of Outcome, Exposure, and Confounding Variables

In this paper we consider particularly respiratory and mental ill-health, as reported by the firefighter. Using visual analogue scales, we asked the firefighter at recruitment to report how much they were bothered by five symptoms (cough, phlegm. breathlessness, wheezing, and chest tightness) before the fire, immediately after their last deployment and at the time of completing the recruitment questionnaire. We then repeated this set of scales at both the first and second follow-up, and used the responses (from 0 to 100) as continuous variables. At the second follow-up participants also completed the ECHRS. Using the approach of Sunyer et al. (18) we had extracted 4 factors (8, 12) we labelled as reflecting cough, phlegm, asthma and wheeze. We also asked if they had any ongoing lung or breathing problems related to the fire.

We screened for mental ill-health with the Hospital Anxiety and Depression Scale (HADS), completed at recruitment and again at first and second follow-up. This gave a score from 0 to 21 on each scale which we could use to look at changes over time. At the second follow-up we also asked the firefighters to complete a 20-item screening questionnaire (the PCL-5) for PTSD, which resulted in a score from 0 to 80. From our psychiatric interview assessment (9), we were able to determine cut-off points for each of the scales that indicated, in these firefighters, those at risk of clinically significant mental ill-health. For anxiety the cut point indicating “caseness” was 12 or greater, for depression 11 or greater and for PTSD, 16 or greater. We also examined mental health diagnoses in physician billing records in the 2 years from May 2nd 2016–March 2018, with any record of mental ill-health (ICD-9 codes 290-319, ICD-10 codes F00-F99.9) being taken as an additional indicator of post-fire mental ill-health.

The calculation of estimated exposure to particulates is explained in Appendix 1. It used information from the firefighter about dates of deployment, shift hours, tasks and geographic location and incorporated objective data from environmental monitoring stations and, for the wildland firefighters, satellite imagery. The Appendix also gives details on how an exposure mitigation index (EMI) was calculated, to reflect the extent to which reported use and type of respiratory protective equipment might be expected to reduce the inhalation of smoke particles. We also considered here the effect on health of the experiences reported by the firefighters. We took the answers to an open-ended question in which we asked the firefighter to “describe your worst moment while working on the Fort McMurray/Horse River fire” and developed a scheme to classify the answers (Appendix 2).

Important potential confounders, in addition to age, sex and, for respiratory disease, cigarette smoking, were the history of respiratory disease and mental ill-health before the fire. For respiratory health, we took a marker for chronic asthma and COPD developed by Alberta Health and included in the information obtained from data linkage. We supplemented this with a report from the firefighter of asthma before the fire. For mental ill-health before the fire we took any diagnosis of mental ill-health (ICD-9 codes 290-319, ICD-10 codes F00-F99.9) in physician billing records from April 1st 2013 to May 1st 2016.

### Statistical Methods

We examined the relation of respiratory complaints at different time points to the composite estimate of exposure to PM_2.5_ particles in a linear regression or, for the report of ongoing respiratory problems, logistic regression. We then carried out a multivariate analysis, adjusting for confounding and, for visual analogue scores of symptoms, reported values immediately prior to the fire. We divided the composite exposure index into two components, overall particulate exposure and the estimated mitigation from RPE and included both in the regression models with respiratory symptoms as the dependent variable. We use conditional logistic regression to analyse the community-based case-referent study, computing odds ratios for mental ill-health before and after the fire and for new onset mental ill-health for those without a mental ill-health diagnosis in the 3 years before the fire. We examined the bivariate relation between estimated particulate exposure (representing the ferocity of the fire) and mental health outcomes both as continuous screening scores (in a linear regression) and “caseness” by logistic regression. We then examined if the firefighters' perceptions of psychological stressors during the fire reflected their mental ill-health before the fire or related to mental ill-health post fire, having adjusted, in a multivariate logistic regression, for mental ill-health before the fire and estimated particulate exposure during the fire. We examined changes of scores on the anxiety and depression scales over time in a multilevel regression analysis and tested, in linear regression models, whether reports of specific “worst moments” added to the model. The analysis was carried out in Stata 14.2.

## Results

**Figure 2:**
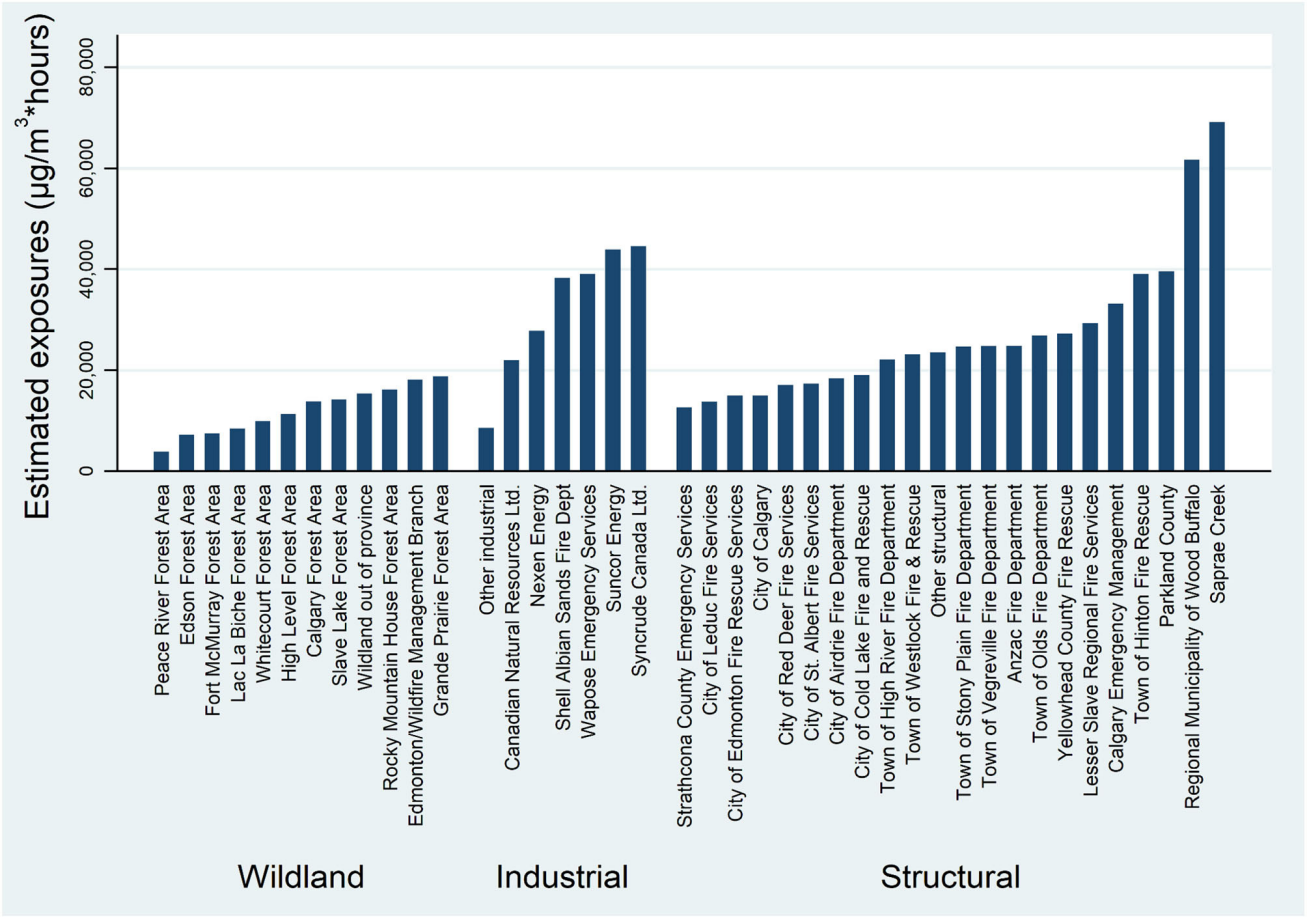
Estimated exposure to PM_2.5_ in firsif00t rotation by fire service and grouped by type of service.

In all, 1,234 firefighters joined the study and completed at least the recruitment questionnaire. Among these were 749 structural, 329 wildland, and 156 industrial firefighters. Participation rates at each phase are shown in Figure 1. Characteristics of the three groups are shown in Table 1. The contract type was asked only in the first follow-up, for which the response rate was relatively low (as those recruited close to the first follow-up were not approached). Where the contract type was not known this was inferred from information from the fire chief for structural and industry-based fire services, with the firefighter assumed to be full-time if the fire chief indicated the majority of fire fighters were full-time and on call/volunteer if that type of contact was the majority for that service. For wildland firefighters all those recruited late (during start-of-season base camps) were assumed to be seasonal workers. There were important differences between the groups of firefighters, with wildland firefighters being younger and more likely to be female, industrial firefighters more likely to be smokers and aged over 40 years and structural firefighters most likely to do only one rotation at the fire. There was, however, considerable variation within the structural fire services, with those working for the regional municipality of Wood Buffalo/Fort McMurray (the Fort McMurray fire service) doing many rotations but firefighters from large city-based fire services generally doing only a single deployment on a rapid-rotation schedule whereby each firefighter spent only 2–3 days at the fire. Mean estimated exposures to PM_2.5_ particles for individual fire services deploying 5 or more firefighters are shown for the key (first) rotation in Figure 2. The estimates for wildland firefighters were derived from satellite imagery (see Appendix 1) rather than land-based monitoring: side by side estimates suggested that the satellite-based estimates were systematically lower than those based on terrestrial air monitoring and the wildland exposures shown in Figure 2 may be underestimated. Among the structural and industrial firefighters, those based in and around Fort McMurray, who were in the midst of the fire right from the start, had the highest exposures.

**Table 1:** Characteristics of firefighters deployed to the fire.

	**Structural**		**Industry**		**Wildland**		**Overall**	
	***N***	**%**	***N***	**%**	***N***	**%**	***N***	**%**
Contract type (^*^assumed)								
Full time	548	73.0	142	92.2	78	23.7	768	62.2
Paid on call/volunteer/seasonal	170	22.6	6	3.9	220	66.9	396	32.1
Non-firefighter role	33	4.4	6	3.9	–	–	39	3.2
Other (e.g., Employed through contractor)	–	–	–	–	31	9.4	31	2.5
**Based in area of Fort McMurray**								
No	602	80.2	–	–	276	83.9	878	71.2
Yes	149	19.8	154	100.0	5.3	16.1	356	28.8
**Repeat rotation**								
One only	537	71.5	58	37.7	163	49.6	758	61.4
2 or more	214	28.5	96	62.3	166	50.4	476	38.6
**Sex**								
Male	693	92.3	147	95.5	267	81.2	1,107	89.7
Female	58	7.3	7	4.5	62	18.8	127	10.3
**Ever smoker**								
No	595	79.2	87	56.5	215	65.3	897	72.7
Yes	141	18.8	61	39.6	98	29.8	300	24.3
Unknown	15	2.0	6	3.9	16	4.9	37	3.0
**Age (years)**								
18–30	199	26.5	31	20.1	178	54.1	408	33.1
31–40	278	37.0	52	33.8	61	18.5	391	31.7
>40	274	36.5	71	46.1	90	27.4	435	35.3
Overall	751	100.0	154	100.0	329	100.0	1,234	100.0

* *Where missing deduced from other information (see text)*.

In Table 2, we examine the relation between estimated exposure and respiratory symptoms, immediately after the fire and at the first and second follow-up. Using visual analogue scales, we asked the firefighter to rate, at the recruitment questionnaire, how troublesome symptoms had been before the fire (not shown) and how troublesome immediately after the fire. We asked them to record these also at the time of first follow-up in 2017–18 and the second follow-up in 2018–19. As can be seen in Table 2, the extent to which they were bothered by each symptom immediately after the fire was related to exposure, but this relationship was less evident by the time of the first follow-up, and by the second follow-up only cough and wheezing were related to exposure. At the second follow-up participants also completed the ECHRS. We had extracted 4 factors of which only one (wheeze) was related to exposure during the fire. We also asked if they had any on-going lung or breathing problems related to the fire. Those who reported that they did (15.8%) had significantly higher estimated exposure.

**Table 2:** Relation of estimated exposure to respiratory symptoms and complaints.

				**Unadjusted**		
**Visual analogue “troublesomeness of symptoms”**	**mean**	**SD**	**N**	**β**	**95% CI**	***P*=**
**After final deployment**						
Cough	33.77	32.64	1,012	6.28	5.09 to 7.47	<0.001
Phlegm	25.76	29.81	1,011	4.37	3.23 to 5.48	<0.001
Breathlessness	18.13	25.33	1,010	4.11	3.17 to 5.05	<0.001
Wheezing	16.95	25.86	1,010	3.65	2.68 to 4.62	<0.001
Chest tightness	16.91	25.33	1,010	3.88	2.93 to 4.82	<0.001
**At 2017–18 follow-up**						
Cough	14.51	20.74	837	1.07	0.20 to 1.94	0.016
Phlegm	15.02	21.59	836	0.31	−0.60 to 1.23	0.499
Breathlessness	9.93	16.39	834	0.96	0.27–1.64	0.007
Wheezing	9.24	16.01	834	0.67	−0.01 to 1.34	0.052
Chest tightness	8.41	15.17	835	1.16	0.52–1.79	<0.001
**At 2018/19 follow-up**						
Cough	15.92	20.71	1,000	0.85	0.07 to 1.62	0.033
Phlegm	15.46	21.97	999	0.10	−0.72 to 0.93	0.804
Breathlessness	11.13	18.39	999	0.50	−0.19 to 1.20	0.153
Wheezing	10.15	17.70	999	0.70	0.03 to 1.36	0.039
Chest tightness	9.68	16.94	999	0.29	−0.35 to 0.93	0.370
**Factors extracted from the ECHRS questionnaire (2018–19)**						
Phlegm	0.00	1.00	995	0.00	−0.04 to 0.04	0.983
Cough	0.00	1.00	995	0.01	−0.03 to 0.05	0.494
Asthma	0.00	0.99	995	−0.00	−0.04 to 0.03	0.807
Wheeze	0.00	0.94	995	0.05	0.01 to 0.08	0.011
			**%**	**N**	**OR**	**95% CI**
**Complaint of lungs or breathing problems related to the Fort McMurray Fire (2018–19)**						
No		84.2	910	1.00	–	–
Yes		15.8	171	1.27	1.13 to 1.42	<0.001
All		100.0	1,081			

The exposure measure used in Table 2 was a composite (Appendix 1) of estimated particulate exposure (from monitoring stations or satellite images) for the dates and locations worked, adjusted by total hours worked on each day, the type of task they were doing and an exposure mitigation index (EMI) reflecting the type of RPE, if any, they reported using, the proportion of time they wore it and the frequency of changing masks or filters. If they wore no RPE the mitigation factor was 1. Sustained use of appropriate equipment reduced the EMI, with a calculated mitigation factor of 0.5 reducing the estimated exposure by 50%. Overall 30% of structural and industrial firefighters wore no RPE. None of the wildland firefighters did so. Among those that did, only 12.5% of structural firefighters and 18.5% of industrial firefighters achieved an EMI of 0.5. To examine the effects of wearing RPE we included everyone as a “wearer” who mitigated their exposure by at least 10%. This accounted for 47% of structural and 44% of industrial firefighters. Among structural firefighters the proportion was lower in those first deployed in the early days of the fire (to May 10th), when only 43% achieved 10% mitigation. In Table 3, we examined further the relation between exposure and respiratory symptoms, adjusting for potential confounders and pre-existing conditions, and partitioning out estimated exposure and the use of RPE. The relation of exposure to symptoms recorded immediately post-fire remained strong, with some mitigation of symptoms of breathlessness, wheezing and, particularly chest tightness amongst “wearers” of RPE. At first follow-up the relation of exposure to symptoms was largely unchanged, with, as before, all but phlegm showing some relation. The coefficient associated with RPE use remained negative (indicating protection) but could be seen as protective only for cough and, weakly, for chest tightness. By the time of the second follow-up, none of the individual symptoms were related to exposure (after adjustment for other factors). The coefficients associated with RPE remained negative, with cough again less in RPE wearers. Among the factors extracted from the ECHRS wheeze remained related to exposure, but not to RPE use. On these scores, cough and asthma appeared to be lower in wearers. The strong relation of ongoing lung or breathing problems to exposure remained after adjustment, with no mitigation by use of RPE.

**Table 3:** Relation of estimated exposure and use of respiratory protective equipment (RPE) to respiratory symptoms and complaints, adjusted^*^.

	**Exposure**			**RPE used**		
**Visual analogue “troublesomeness of symptoms”**	**β**	**95%CI**	***P*=**	**β**	**95% CI**	***P*=**
**After final deployment**						
Cough	6.34	5.16 to 7.33	<0.001	−2.81	−6.70 to 1.09	0.157
Phlegm	4.67	3.62 to 5.72	<0.001	−0.88	−4.33 to 2.57	0.617
Breathlessness	4.13	3.23 to 5.02	<0.001	−2.82	−5.78 to 0.14	0.062
Wheezing	3.66	2.73 to 4.59	<0.001	−2.70	−5.77 to 0.37	0.084
Chest tightness	4.01	3.11 to 4.92	<0.001	−3.13	−6.10 to 0.15	0.039
**At 2017–18 follow-up**						
Cough	1.22	0.33 to 2.10	0.007	−3.38	−0.63 to 0.46	0.023
Phlegm	0.61	−0.31 to 1.53	0.196	−1.34	−4.37 to 1.70	0.388
Breathlessness	0.82	0.15 to 1.49	0.017	−1.36	−3.57 to 0.84	0.225
Wheezing	0.62	−0.05 to 1.29	0.069	−0.29	−2.49 to 1.91	0.798
Chest tightness	1.18	0.55 to 1.82	<0.001	−1.82	−3.91 to 0.26	0.087
**At 2018/19 follow-up**						
Cough	0.69	−0.21 to 1.59	0.135	−3.63	−6.56 to 0.71	0.015
Phlegm	−0.05	−0.99 to 0.90	0.922	−2.67	−5.74 to 0.39	0.087
Breathlessness	0.45	−0.35 to 1.25	0.269	−1.46	−4.07 to 1.14	0.271
Wheezing	0.48	−0.26 to 1.21	0.203	−0.74	−3.13 to 1.66	0.547
Chest tightness	0.18	−0.55 to 0.90	0.634	−1.77	−4.14 to 0.57	0.139
**Factors extracted from the ECHRS questionnaire**						
Phlegm	−0.00	−0.04 to 0.04	0.986	0.02	−0.11 to 0.15	0.781
Cough	0.01	−0.03 to 0.05	0.630	−0.16	−0.29 to 0.02	0.021
Asthma	−0.00	−0.03 to 0.03	0.950	−0.11	−0.21 to 0.00	0.040
Wheeze	0.04	0.01 to 0.08	0.015	−0.05	−0.18 to 0.07	0.393
	**OR**	**95%CI**	**P=**	**OR**	**95%CI**	**P=**
**Complaint of lungs or breathing problems related to the Fort McMurray Fire (2018–19)**						
No	1.00	–		1.00	–	
Yes	1.30	1.15 to 1.46	<0.001	0.92	0.65 to 1.31	0.648
All						

* *Adjusted for smoking, sex, age at deployment, asthma prior to the fire, and for visual nscores, rating of troublesomeness on that dimension prior to the fire*.

Two supplementary analyses were carried out. First, we examined the effect of wearing RPE just in those who had been deployed during the first week of the fire, where almost every task involved high exposures. Later in the fire those patrolling, for example, did not necessarily need RPE. For breathlessness, wheezing and chest tightness immediately postfire the effects of RPE were seen only in those deployed during the first week (data not shown). Similarly, at the first and second follow-up the mitigating effect of RPE on cough was only in those deployed early. Second, we looked to see if we could establish a degree of mitigation that showed effects across all, or most, end points, and particularly that of ongoing lung problems. We did not find evidence of such a mitigation point and no fewer problems were seen in the small group whose exposure was estimated to be mitigated by 50% or greater.

A case-referent analysis of mental ill-health in firefighters compared with the matched community controls, in a conditional logistic regression, showed little effect of the fire (Table 4). Before the fire, somewhat fewer firefighters than community controls had a mental health diagnosis in physician billing records (OR = 0.84). This difference was smaller in the period after the fire (OR = 0.91) but there was no marked increase in the diagnosis of new onset mental ill-health in firefighters (OR = 1.09) in physician billing records. The number of diagnoses of PTSD recorded in billing records was very small (17 overall, 8 in firefighters, 9 in controls) but the odds of a PTSD diagnosis being recorded were higher in firefighters (OR = 4.47 95% CI 1.72–11.62 *p* = 0.002).

**Table 4:** Case referent analysis of mental ill-health^*^ as recorded in the Alberta administrative health database.

		**Any mental ill-health from April**				**Any mental ill-health from May**				**New onset mental ill-health from May**			
		**1st 2013 to May 1st 2016**				**2nd 2016 to March 30th 2018**				**2nd 2016 to March 30th 2018**			
	***N***	***n***	**%**	**OR**	**95% CI**	***N***	**%**	**OR**	**95% CI**	***n***	**%**	**OR**	**95% CI**
Firefighters	955	242	25.3	0.84	0.71–0.99	235	24.6	0.91	0.77–1.07	110	11.5	1.09	0.88–1.36
Community controls	4,775	1,362	28,5	1	–	1,260	26.4	1	-	508	10.6	1	–
All	5,730	424	7.4	–	–	312	5.4	–	–	126	2.2	–	–

* *ICD-9 code 290-319; ICD-10 code F00-F99*.

We considered next the relation of mental ill-health following the fire to estimated exposure to particulate matter during the fire (Table 5). We examined this in relation to “caseness,” 2 years or more after the fire and to the anxiety and depression scores of the Hospital Anxiety and Depression Scale, completed on three occasions, at recruitment, and at first and second follow-up. Mental ill-health from physician records was not related to particulate exposure but the risk (odds ratio) for caseness, using definitions from screening scores, increased with increasing exposure during the fire, less strongly for depression than for anxiety or PTSD. The relation to exposure decreased with time since the fire, particularly for anxiety.

**Table 5:** Bivariate relation of particulate exposure during the fire to mental health post-fire.

	**OR**	**95% CI**	***P*=**	***N***
**(A) “Caseness” 2 years or more post-fire (logistic regression)**				
Mental ill-health in billing records	1.07	−0.98 to 1.18	0.119	955
Anxiety ≥ 12	1.32	1.14 to 1.52	<0.001	1,000
Depression ≥ 11	1.23	1.00 to 1.50	0.046	1,000
PTSD ≥ 16	1.34	1.19 to 1.52	<0.001	998
	**β**	**95% CI**	***P*** **=**	***N***
**(B) Anxiety and depression as continuous scores by contact post-fire (linear regression)**				
Recruitment				
Anxiety	0.41	0.27 to 0.56	<0.001	1,019
Depression	0.26	0.15 to 0.37	<0.001	1,019
First follow-up				
Anxiety	0.25	0.09 to 0.42	0.002	831
Depression	0.13	0.02 to 2.91	0.055	831
Second follow-up				
Anxiety	0.28	0.13 to 0.43	<0.001	1,000
Depression	0.24	0.11 to 0.37	<0.001	1,000

We next looked to see whether the firefighters' own reports of their “worst moment during the fire” recorded on the recruitment questionnaire was related to their mental health. The open-ended responses were coded into 10 categories (Appendix 2), five reflecting psychological stressors and five physical ones. Many responses were a composite, such as sleep deprivation and smoke or devastation and noise such that a firefighter's response could receive multiple codes. Overall, 48% gave at least one worst moment coded as “psychological” and 42% one coded as “physical”: 24% did not write anything or wrote “no worst moment.” Responses referring to seeing/experiencing the devastation caused by the fire were most common, closely followed by references to the dense smoke. In Table 6 we show these frequencies for all respondents and also broken down by the presence of mental-ill health in physician billing records in the 3 years prior to the fire. Those with such a pre-fire diagnosis were no more likely to report psychological stressors as their “worst moment” during the fire. The relation of each element coded as a worst moment to “caseness” is shown in Table 7. We carried out a logistic regression analysis for each stressor in turn, and for the total number of psychological and physical stressors recorded. Each regression was adjusted to allow for mental ill-health before the fire, for the concentration of smoke particles (reflecting the intensity of the fire) and for sex and age. Mental ill-health from billing records post-fire was related only to reports of relational stress (involving worries about family, animals, homes, or communities to which they have a specific attachment–Appendix 2). With caseness defined from screening scores, anxiety, and PTSD were more likely in those reporting multiple psychological stressors and all three were more likely in those who were in a situation where they perceived a threat to their life or safety.

**Table 6:** Reports of “worst moment” during the fire by mental health before the fire (from physician billing records).

**Nature of “worst moment” reported**	**No**		**Yes**		**Unknown**		**Overall**		P^*^ =
	***n***	**%**	***n***	**%**	***n***	**%**	***N***	**%**	
**Mental ill-health prior to the fire**									
(A) Psychological									
Psychological/mental strain	92	15.0	25	12.3	32	16.5	149	14.8	0.468
Relational	56	9.2	11	5.4	15	7.7	82	8.1	0.229
Inter crew	60	9.8	22	10.8	16	8.2	98	9.7	0.688
Devastation	130	21.2	53	26.0	37	19.1	220	21.8	0.217
Threat	69	11.3	24	11.8	10	5.2	103	10.2	0.035
(B) Physical									
Smoke	105	17.2	44	21.6	37	19.1	186	18.4	0.359
Exhaustion	58	9.5	20	9.8	20	10.3	98	9.7	0.942
Sleep deprivation	59	9.6	21	10.3	21	10.8	101	10.0	0.881
Physical stress	60	9.8	22	10.8	30	15.5	112	11.1	0.090
Lack of resources	73	11.9	26	12.7	13	6.7	112	11.1	0.091
Number of psychological									
0	313	51.1	99	48.5	113	58.2	525	52.0	0.412
1	211	34.5	80	39.2	56	28.9	347	34.4	
2	69	11.3	20	9.8	21	10.8	110	10.9	
3–5	19	3.1	5	2.5	4	2.1	28	2.8	
Number of physical									
0	360	58.8	119	58.3	109	56.2	588	58.2	0.526
1	162	26.5	44	21.6	54	27.8	260	25.7	
2	79	12.9	35	17.2	26	13.4	140	13.9	
3–5	11	1.8	6	2.9	5	2.6	22	2.2	
*N*	612	100.0	204	100.0	194	100.0	1,010	100.0	-

* *chi square*.

**Table 7:** Relation of mental ill health 2 years or more post fire to “worst moment.”

**Nature of “worst**	**Mental ill-health from**			**Anxiety score > 12**			**Depression score ≥ 11**			**PTSD score ≥ 16**		
**moment” reported**	**billing records post fire**											
	**OR**	**95% CI**	***P*=**	**OR**	**95% CI**	***P*=**	**OR**	**95% CI**	***P*=**	**OR**	**95% CI**	***P*=**
(A) Psychological												
Psych/mental stress	0.98	0.60–1.61	0.951	1.15	0.66–2.00	0.631	1.68	0.79–3.58	0.178	1.93	1.23–3.03	0.004
Relational	2.08	1.18–3.70	0.012	0.99	0.47–2.11	0.987	0.52	0.12–2.22	0.376	1.28	0.70–2.36	0.421
Inter crew	1.28	0.75–2.20	0.370	2.07	1.16–3.69	0.014	1.36	0.55–3.36	0.505	1.67	0.98–2.83	0.057
Devastation	0.93	0.62–1.40	0.717	1.22	0.76–1.97	0.408	1.38	0.70–2.72	0.352	1.95	1.32–2.88	0.001
Threat	0.93	0.54-1.60	0.796	2.06	1.17-3.63	0.012	2.57	1.19-5.53	0.016	1.98	1.20-3.29	0.008
(B) Physical												
Smoke	1.41	0.93–2.13	0.107	1.33	0.80–2.21	0.267	2.06	1.06–3.99	0.032	0.99	0.63–1.55	0.951
Exhaustion	0.49	0.25–0.95	0.034	0.68	0.31–1.47	0.326	0.65	0.20–2.18	0.489	0.48	0.23–1.00	0.049
Sleep deprivation	0.98	0.56–1.72	0.945	0.89	0.42–1.86	0.753	1.32	0.50–3.47	0.579	0.80	0.42–1.53	0.498
Physical stress	1.06	0.60–1.86	0.582	1.17	0.61–2.27	0.633	1.15	0.44–3.02	0.777	0.63	0.32–1.24	0.183
Lack of resources	1.37	0.84–2.26	0.210	1.95	1.12–3.41	0.019	1.71	0.76–3.86	0.196	1.14	0.67–1.95	0.631
Number of psychological	1.10	0.88–1.38	0.388	1.42	1.10–1.83	0.007	1.42	0.99–2.04	0.056	1.76	1.10–1.45	<0.001
Number of physical	1.04	0.84–1.28	0.750	1.10	0.86–1.42	0.448	1.24	0.87–1.76	0.231	0.82	0.65–1.04	0.105
*N*	881			840			840			838		

Logistic regression analysis*.

*Adjusted for mental ill health from billing records before the fire, estimated exposure to particles during the fire, sex and age.

We examined scores on the anxiety and depression scales over time (Table 8) and by the record of mental ill-health before the fire. At each point anxiety and depression scores were higher in those with pre-fire mental ill-health. Examination of the overall mean scores suggested that both anxiety and depression scores increased with time since the fire, with scores at second follow-up higher than at recruitment. In a series of regression analyses with score at second follow-up as the dependent variable and with score at recruitment also in the model, we examined whether any of the reported “worst moments” appeared to accelerate or reduce this deterioration in mental health. Increase in anxiety was less in those who were older at recruitment and was unrelated to gender. Those with a pre-fire history of mental ill-health had a greater increase in anxiety than those without. From the worst moment data, only the perception of threat to life or safety was a predictor of greater increase in anxiety (Table 9). A parallel analysis for depression scores showed no relation to age or gender but again those with a previous history of mental ill-health had a greater increase in depression score. None of the psychological worst moments was related to the increase in depression but both the report of dense smoke and sleep deprivation were associated, with a final multivariate model (Table 9) including only sleep deprivation. We also conducted a parallel analysis stratifying by previous mental ill-health to see if those with such a history were particularly vulnerable to specific stressors. With this smaller group the power of the analysis to detect differences was lower but we saw increased anxiety at second follow-up in those who reported at recruitment that there had been difficulties in interpersonal relation with their crew during the fire (Table 9). Estimated exposure was not related to deterioration in mental ill-health between recruitment and 2nd follow-up and did not add to the models in Table 9.

**Table 8:** Mean anxiety and depression scores by mental ill health before the fire, at recruitment and at each follow-up.

	**Anxiety**						**Depression**					
	**Mental ill health before the fire**				***F***	***P***	**Mental ill health before the fire**				***F***	***P***
**Data point**	**No**	**Yes**	**Unknown**	**Overall**			**No**	**Yes**	**Unknown**	**Overall**		
**Recruitment**												
Mean	4.51	5.41	4.50	4.69	4.45	0.012	2.28	2.90	2.59	2.47	3.70	0.025
SD	3.71	4.23	3.97	3.88			2.76	3.28	2.94	2.92		
N	616	207	196	1,019			616	207	196	1,019		
**First follow-up**												
Mean	4.69	6.21	4.99	5.05	9.85	<0.001	2.62	3.45	3.05	2.86	4.41	0.012
SD	3.68	4.21	3.86	3.86			3.09	3.57	3.26	3.23		
N	517	164	150	831			517	164	150	831		
**Second follow-up**												
Mean	5.59	6.92	5.96	5.92	7.97	<0.001	3.19	4.06	3.89	3.49	6.43	0.002
SD	3.95	4.43	4.08	4.10			3.23	3.87	3.57	3.45		
N	613	197	190	1,000			613	197	190	1,000		
*P*=												
**Recruitment to**												
1st follow-up	0.311	0.007	0.151	0.008			0.005	0.021	0.071	<0.001		
2nd follow-up	<0.001	<0.001	<0.001	<0.001			<0.001	<0.001	<0.001	<0.001		

**Table 9:** Final models for anxiety and depression at second follow-up, with worst moment allowing for anxiety or depression score at recruitment.

	**Anxiety**						**Depression**		
	**All**			**Just with prior mental ill health**			**All**		
	**β**	**95% CI**	***P*=**	**β**	**95% CI**	***P*=**	**β**	**95% CI**	***P*=**
Mental ill-health before the fire									
Yes	0.75	0.20 to 1.29	0.007				0.51	0.04 to 0.97	0.033
Unknown	0.14	−0.45 to 0.72	0.647				0.30	−0.20 to 0.51	0.240
Score at recruitment	0.69	0.63 to 0.74	<0.001	0.66	0.53 to 0.79	<0.001	0.78	0.71 to 0.84	<0.001
Age at recruitment	−0.03	−0.05 to 0.01	0.002				–	–	–
Worst moment									
Threat to life/well-being	0.77	0.70 to 1.46	0.031	–	–	–	–	–	–
Inter-crew difficulties	–	–	–	1.79	0.12 to 3.46	0.036	–	–	–
Sleep deprivation	–	–	–	–	–	–	0.80	0.16 to 1.43	0.014
Constant	3.66	2.80 to 4.51	<0.001	4.22	2.03 to 6.41	<0.001	1.48	1.20 to 1.75	<0.001
*N*	837	169	837						

Finally, we looked at exposures during the fire and previous ill-health in those who had a fire-related injury claim accepted by the Workers Compensation Board (WCB). Among 882 firefighters who agreed that we might access information from their WCB records, only 17 had an accepted compensation claim for respiratory ill-health and 37 for mental ill-health. Those compensated for either respiratory or mental ill-heath had higher particulate exposure than those who did not have such a claim. Those compensated for respiratory ill-health were no more likely to have a pre-fire history of chronic lung disease or to have been a smoker, but the 20 firefighters compensated for PTSD were more likely than those without an accepted claim to have had a mental-ill health diagnosis in the physician billing record in the 3 years before the fire. Less than half of those compensated for any psychological injury, and only 59% of those compensated for PTSD, had a physician billing diagnosis for mental ill-health in the 23 months after the start of the fire.

## Discussion

This paper has considered reports from the firefighters themselves on respiratory and mental ill-health in the years since the start of the Fort McMurray/Horse River fire. Respiratory symptoms were overall less marked with increasing time since the fire, with little relationship to estimated particulate exposure at the second follow-up, some 30 months after the start of the fire. The subgroup of 16% of firefighters reporting on-going lung or breathing problems related to the fire were drawn from those more heavily exposed and, as has been shown elsewhere (12), had an increased risk of clinically verified airways hyperreactivity and bronchial wall thickening. Mental ill-health, as reflected in the anxiety and depression scales of the HADS, showed a rather different pattern, with increased anxiety and depression with longer time since the fire. At each contact, scores related to the intensity of exposure during the fire, but that relationship was stronger immediately post-fire. Those reporting at recruitment that they felt their life or safety had been under threat during the fire were more likely to have high scores, indicating risk of clinically significant mental ill-health, at second follow-up. As has been reported elsewhere (9) the prevalence of mental ill-health, estimated from structured clinical interviews, was high in this cohort (21% PTSD 16% anxiety disorders 14% depressive disorders). The increasing anxiety and depression scores with time since the fire might simply reflect a learning effect (with higher scoring as the scale became more familiar) but equally might reflect in part unresolved issues arising from the fire or the ongoing stresses of working as a first responder (19). The responses given during the structured clinical interview to assess PTSD (9) showed clearly that fighting fires was not the only, or necessarily the major, cause of PTSD. Only 42% of those with PTSD reported during the clinical interview any fire event as life threatening but cited rather the trauma of dealing with the dead and injured in road traffic and other incidents (20).

A strength of this study is its longitudinal follow-up, which allows us to look at the evolution and resolution of fire-associated symptoms. We were fortunate also to be able to link firefighters to their administrative health record and so have some indication of their pre-fire health and how this compared to others in their community. This was particularly valuable looking at respiratory ill-health where we found an excess of asthma in firefighters pre-fire with increased numbers post-fire (12). A comparable analysis for mental ill-health, included here, was less successful in demonstrating a post-fire excess in firefighters largely, perhaps, because assessment or treatment by psychologists would not be reflected in this database (but was almost certainly part of the assessment in those with WCB compensation).

Weaknesses of the study include missing or imputed values for some wildland firefighters, many of whom were recruited some months after the fire, completed only a short recruitment questionnaire and were not asked to complete the first follow-up questionnaire. Not everyone agreed to be linked to the AHDB or to WCB records, and those who did may not be representative of the whole sample. Use of the physician billing records to reflect mental ill-health before or after the fire will have excluded mental ill-health assessed by other health professionals. The effectiveness of RPE was by an index that has not been objectively validated.

There have been many studies of the respiratory and mental ill health of firefighters, with systematic reviews concluding that the risk of ill-health was greater after large scale disasters (4, 6). Longitudinal studies, following up the health of firefighters in the months and years after the fire are uncommon. Those of mental health in Australian firefighters following disastrous bush fire in 1983 deserve mention (21) but the prototype for the current study was the longitudinal follow-up of first responders from the collapse of the World Trade Centre in 2001. As here, increases in asthma, airways hyperreactivity, PTSD, depression and stress reactions were well-documented (22). Although the exposures were different, with particulate exposure in the early hours being to dust clouds rather than smoke, and horrendous loss of life in the World Trade Centre disaster, the similarity in health outcomes is striking.

In setting up this study we wanted to help identify ways in which fighting future wild fires might be made safer. We have shown evidence here of the very limited respiratory protection achieved by RPE, particularly in the early days of the fire. Elsewhere we have shown the importance of skin hygiene to reduce absorption of polycyclic aromatic hydrocarbons both in urban and industrial firefighters in the Fort McMurray fire (7) and in wildland firefighters (23). The importance of administrative controls, in reducing exposures by deploying firefighters in rapidly rotating shifts, is demonstrated by the mean exposures during the first rotation shown in Figure 2. Firefighters from Strathcona and the City of Edmonton, for example, attended the fire from the earliest days alongside those from Fort McMurray/Wood Buffalo, but because of the short time each firefighter was deployed, the mean exposure was among the lowest for structural firefighters. Through our study of fire chiefs and their reports of mental health supports, we have shown also that anxiety and depression, and perhaps PTSD, were reduced by peer support provided after the fire (11). The result from the current analysis, showing that those who felt that their life or safety had been threatened during the fire had increasing anxiety with time since the fire, reinforces the need for ongoing support from peers and health professionals. Work on this cohort has also highlighted the link between difficult childhood circumstances and psychiatric ill-health suggesting the importance of trauma-informed mental health care in these first responders (10, 24).

Although the main aim of the study was to help identify approaches to the primary prevention of harm to firefighters, a secondary goal was to use the data to assist in determining whether damage to firefighters' health was indeed fire-related. In one paper (8) we examined the relation of inflammatory markers, taken 3–4 months after the start of the fire, to both exposure and respiratory ill-health and suggested that, in the absence of external estimates of exposure, long term biomarkers might be a useful proxy. From the clinical assessment of those with ongoing respiratory problems, we suggest that the combination of airways hyperreactivity and bronchial wall thickening might be used as a marker of fire-related damage (12).

We have been invited to consider, in this paper, how rapid research could improve health outcomes and resilience following a disaster such as the fire in Fort McMurray. We have learnt that the collection of data and samples, particularly biological exposure markers, as early as can possibly be achieved, may be critical in establishing the full effects of events and exposures that occur before control of the disaster site has been fully established (25). This may be very difficult, given the need to limit access to the site, but may be possible with collaboration from those within the perimeter. A second need is to establish a nominal list of participants before they are too widely dispersed. In this emergency we were particularly fortunate in having near instant support from potential collaborators, funders and the university ethics board. Serendipitously we had available the mobile clinical laboratory and access to detailed lists of firefighters. By getting on the ground early we were able to establish credibility with firefighters who could help us devise questions appropriate to their experiences. By establishing an Investigators Group, with representatives from firefighter organisations, we did our best to ensure that each of the follow-up contacts was appropriate and worthwhile. It was particularly valuable to the team to recruit firefighters face-to-face during the first phase of the study, and to hear about their experiences, often troublesome, during the early days of the fire. In this final report we have concentrated on the perceptions of the firefighters themselves: although changes to policies and programs must rest on the strongest and often most objective evidence, full insight into the fire and its effects needed the early and full collaboration of the firefighters, which we have tried to reflect here.

## Data Availability Statement

The raw data supporting the conclusions of this article will be made available by the authors, without undue reservation.

## Ethics Statement

The project was reviewed by the Health Ethics Board at the University of Alberta (Pro00089958 and Pro00098071). The patients/participants provided their written informed consent to participate in this study.

## Author Contributions

NC and JB designed the study and collected the data. NC and J-MG assembled the database and conducted the analysis. NC wrote the first draft of the manuscript. All authors reviewed, revised, and approved the manuscript.

## Funding

This work was funded by the Canadian Institutes for Health Research (FRN151027, 162537) and the Government of Alberta, OHS Futures.

## Conflict of Interest

The authors declare that the research was conducted in the absence of any commercial or financial relationships that could be construed as a potential conflict of interest.

## Publisher's Note

All claims expressed in this article are solely those of the authors and do not necessarily represent those of their affiliated organizations, or those of the publisher, the editors and the reviewers. Any product that may be evaluated in this article, or claim that may be made by its manufacturer, is not guaranteed or endorsed by the publisher.
