# Supplementary material for: The Health of Firefighters Deployed to the Fort McMurray Fire: Lessons Learnt
Source: Front Public Health. 2021 Nov 11;9:692162. doi: 10.3389/fpubh.2021.692162 (PMC8632044; doi:10.3389/fpubh.2021.692162)
Supplement: Supplementary file 1 [file Data_Sheet_1.pdf]

Appendix 1 Estimation of exposure to smoke particulates (total $PM_{2.5}$) and Exposure Mitigation Factor

A) Estimation of environmental smoke concentration (total $PM_{2.5}$) encountered by each firefighter during the key rotation.

Exposures were first estimated for the 'key' deployment. This was the first deployment for all but 4 firefighters from fire station A, who gave information only for their most recent deployment. Elements used for this calculation were as follows:

1) Data available from Alberta Environment and Parks. Daily 24-hour average concentrations of $PM_{2.5}$ ($\mu g/m^3$) for each day from May 1st 2016 to June 30th 2016 were provided for 9 locations within the urban area of Fort McMurray. These had been listed in the questionnaire and the firefighter was requested to indicate the percentage of time spent in each. These were: Timberlea, Thickwood, Abasand, Lower Town Site, Waterways, Beacon Hill, Gregoire, Saline Creek, Parson's Creek. Estimates were also obtained for 3 further locations (the airport, the village of Anzac and the area of Mildred Lake) as these were reported by firefighters as additional areas in which they spent time during their key deployment. All estimates except those for Mildred Lake used data from Alberta Environment monitoring stations. The estimates for Mildred Lake use Blue Sky estimates (a combination of air samples taken at different locations and satellite imagery).
2) Total hours on active duty reported by the fire fighter during the key rotation. The number of hours worked on each day of each shift during the key deployment was calculated. For example: for firefighter 000 shift 1 started at 9h00 on May 3rd with reported length 23 hours; he will have worked from 9h00 to 23h59 on May 3rd so 15 hours for May 3rd and he will have worked 9 hours on May 4th. This was repeated for every shift as to determine how many hours were worked on which days for their entire key deployment.
3) The percentage of time spent in each location during the key deployment (as reported by the firefighter). This was used to compute a time weighted exposure for each firefighter for each day of deployment. For example: firefighter 000 worked on May 3rd 10% in Parson's Creek and 80% in Timberlea for 5 hours. Alberta environment has a 24-hour mean for Parson's Creek on May 3rd of 535 µg/m3 and of 320 for Timberlea (535*5*.1) + (320*5*.8).
4) The total time was used to adjust the time weighted estimate. $PM_{2.5}$ estimates were adjusted to reflect 100% of their day because many firefighters either overestimated or underestimated the amount of time they spent in each location (i.e. the total percentage exceeded or fell short of 100%). Example: (535*5*.1) + (320*5*.8)=1547.5 for 90% of key deployment adjusted as 1547.5/0.9=1719.44.
5) A cumulative exposure for their key deployment was obtained by summing over all shifts.

B) Other Deployments.

For all firefighters who had multiple deployments to the Fort-McMurray fire between May 1st and June 30th 2016, no information was available about where they

had worked or how much time they spent in each area. For these later deployments exposure was estimated from the exposures of firefighters whose key deployment was during this period.

To do this, cumulative estimates were divided into each day worked during their secondary deployments, using the estimates calculated on each day in Step 1. A mean exposure was obtained for every date worked in a key deployment. Data were available for almost every day between May 1st to June 30th. For dates, mostly in June, for which no data were available, data from Alberta Environment and Parks were used to calculate the means for each of those days, assuming a 10 hour working day, the mean number of hours worked by firefighters in late May and June.

This provided a per deployment estimate. These were summed up to obtain a cumulative exposure during other, non-key deployments.

C) Task Factor

1) Self-reports of typical smoke exposure while doing that task, were reported using visual cues published by Reinhardt and Ottmar (2000). The firefighter rated intensity of smoke on a scale from none to very heavy, comparing their exposure to the published pictures . These were weighted using the regression data of exposure to $PM_{3.5}$ measured at the time Reinhardt and Ottmar took the visual cue photographs.
2) For each of up to 9 tasks, the reported proportion of time spent on the task during the key deployment was weighted by the mean smoke intensity score for all doing that task during the key deployment.
3) The smoke intensity scores were then summed for each firefighter giving a total task exposure score for the key deployment.
4) The task exposure score was finally adjusted over the total reported % of time in tasks (i.e. the total percentage exceeded or fell short of 100%).

Reinhardt T, Ottmar R. Smoke exposures at western wildfires. United States Department of Agriculture, Research paper PNW-RP-525 July 2000. Pacific North West Research Station. Accessed at http://depts.washington.edu/wildfire/resources/smokewestern.pdf 5th July 2020.

C) Respiratory protection factor (Exposure Mitigation Index)
Range: 0=best to 1=none

- The reciprocal of the protection factor of the best RPE equipment identified was computed (protection factor of 10=1/10 unprotected).
- The recommended time to change of a cartridge was taken as 5 hours (reflecting a reduction from 8 hours due to heavy exposures) to which the total time spent in each task was divided by 5 to produce a number of times *should have changed*. The time in each task was divided by the number of hours to actual change producing number of times actually changed.

- A ratio of *times changed* to *should have changed* was computed. This ratio was multiplied by the total time wearing a respirator in each task to produce a time improperly wearing RPE which was added to the actual time not wearing RPE (when ratio<1.0). This time was treated as unprotected.
- The reciprocal of the RPE was used and multiplied by the remaining properly protected time.
- Both of these values were added giving us a value between 0 and 1 which represents an unprotected time factor (1=unprotected, 0=100% protected).
- This was cumulated over all tasks and divided by the number of tasks and adjusted to reflect 100% of their time during their key deployment.

E) Final exposure variable; composite of above variables

The key deployment was multiplied by the respiratory protection factor and then by the task factor score and the cumulative exposures estimated for secondary deployments were added. A log transformation was made of the final value. This could be represented by the equation below:

$$composite\ exposure\ E = \log((A * C * D) + B)$$

F) Highest day by area exposure

This represented the highest environmental exposure to $PM_{2.5}$ on any one day in any area worked.

Wildland

Wildland firefighters had essentially the same algorithm applied for their calculation of their exposures with the exception that the daily $PM_{2.5}$ estimates came from Bluesky data which were estimates derived from satellite imagery. Instead of the same locations as for the structural firefighters, their locations were taken from a grid shown on a map colour coded for mean $PM_{2.5}$ levels. When a firefighter indicated spending time in two or more cells on the Bluesky map, these were combined to produce the mean for that day and this was used as the estimate for that day. We also tailored for each firefighter that had a cell identified in our grid but that was not in our predefined options on the questionnaire and added another estimate which was individualized to each “other” cell. For those with missing dates and locations, a buddy system was adopted where an attempt was made at matching the firefighter with missing information to another firefighter in the same role, with a close age and the same sex. The rest of their exposures were calculated in the same way as described above, without adjustment for respiratory protection (not used by wildland firefighters).

Appendix 2: Coding of the 'worst moment'

Firefighters were asked the following question: "Describe your worst moment while working on the Fort McMurray/Horse River fire." We established 10 thematic categories that covered the range of different experiences described by the firefighters.

Once categories were established, the worst moment response was coded one-by-one (yes = 1 or no = 0) on each of the 10 codes (which were not mutually exclusive). If a response was uncodable , had no worse moment, or was left blank it was noted on the coding sheet.

If a response was codable, the 10 categories used to code the firefighters' worst moment during their Fort McMurray deployment included:

1. Psychological/Mental Strain
2. Relational
3. Inter-Crew Relations
4. Devastation
5. Threat
6. Smoke Exposure
7. Exhaustion
8. Sleep Deprivation
9. Physical Strain
10. Resources

A brief definition and description of each category is provided below.

1. **Psychological/Mental Strain**
   All explicit references to psychological and mental strain (e.g. scared) related to the fire were coded under this category. Implied references to psychological strain were also categorized as mental if that strain was directly related to their deployment experience, such as being trapped by fire, feeling the fire was out of control, feeling they were failing, forced to abandon a firefight, or were not deployed enough.

2. Relational
   Relational refers to all 'concern for other' responses about direct (non-crew) relationships. Responses that mentioned concern or worry about one of the following were coded as relational: safety of family or friends, concern for someone's property, worry about evacuation of civilians, and concern for a community that they have a direct attachment too. Generic concerns for the city were not coded under relational.

3. Inter-Crew Relations
   All responses that related to concerns and problems with other firefighters were coded as inter-crew relations. Concern for another crew member included things like worrying about the safety of other crews and the evacuation of firefighters. Problems with other crew members included feeling neglected by firefighters, lack of leadership, poor relationships, and ordering firefighters to abandon

a fire/home. Responses had to imply that the inter-crew relationship was impacted in some way to qualify.

4. **Devastation**
   All responses that refer to the loss or carnage caused by the fire were coded as devastation. Many firefighters used the language of devastation and destruction of the city to describe their experience. Responses that described seeing damage, burning houses/neighbourhoods, or when a firefighter was deployed to an area known to have devastation were also coded under this category. Devastation is a distinct category from threat, which refers to a firefighter who is under immediate threat from an active fire.

5. **Threat**
   Threat refers to direct risk/danger to the firefighter from an active fire. All explicit references to threat or situations that implied threat were coded here, including being trapped by fire, fire being out of control, risky/dangerous fires, or feeling scared for their life from the fire. Simply being near an active fire or heat/flame – without reference to threat – was not coded under this category.

6. **Smoke**
   Smoke exposure was coded as a pure category. Only clear references to smoke – including acid, toxic clouds, ash, inversion and air quality – were coded as smoke. Other related terms like flame, heat and hot spots were not coded as smoke.

7. **Sleep**
   Sleep deprivation was coded as a pure category. Only responses that directly mentioned sleep or closely related terms – like being tired, woken/disrupted from sleep, or needing rest – were coded as sleep. Sleep was coded as a distinct category from exhaustion.

8. **Exhaustion**
   Exhaustion was coded as a pure category. Only response that clearly referred to exhaustion – such as mental/physical fatigue, feeling drained, or references to long days – were categorized here. Exhaustion was coded as a distinct category from sleep.

9. **Physical Strain**
   Physical strain is a broad category that refers to physical body pain (that is not related to mental, smoke, exhaustion or sleep) and physical exertion on the job. Responses coded as physical body pain were wide ranging, including being cold/wet, hot/heat, injuries, sore body parts, dehydration, and high noise. Responses that involved heavy physical exertion were also coded as physical strain, including holding a hose for a long time, digging ash pits, hauling equipment, fast pace of work, overhaul, working in hot spots, and tearing down structures.

10. **Resources**
    Resources were broadly defined as a firefighter lacking equipment to fight fires or the basic

necessities. Lack of equipment ranged from not having exposure protection, respiratory equipment, no or low water pressure, breakdown of equipment, lacking proper firefighter gear to not knowing how to use equipment. Responses were also categorized as resource issues if they mentioned not having basic necessities like food, water, clothes or facilities (shower, sleeping quarters, etc.).

Double Coding

The following responses were always double coded:

- Trapped by fire (psychological and threat)
- Out of control fire (psychological and threat)
- Threat to life (psychological and threat)
- Mental fatigue/exhaustion (psychological and exhaustion)
- Physical fatigue/exhaustion (physical strain and exhaustion)
